# Management of anastomotic leakage after robot-assisted minimally invasive esophagectomy with an intrathoracic anastomosis

**DOI:** 10.1093/dote/doac094

**Published:** 2023-01-12

**Authors:** Eline M de Groot, Sebastiaan F C Bronzwaer, Lucas Goense, B Feike Kingma, Sylvia van der Horst, Jan Willem van den Berg, Jelle P Ruurda, Richard van Hillegersberg

**Affiliations:** Department of Surgery, University Medical Center Utrecht, Heidelberglaan 100, Utrecht, The Netherlands; Department of Surgery, University Medical Center Utrecht, Heidelberglaan 100, Utrecht, The Netherlands; Department of Surgery, University Medical Center Utrecht, Heidelberglaan 100, Utrecht, The Netherlands; Department of Surgery, University Medical Center Utrecht, Heidelberglaan 100, Utrecht, The Netherlands; Department of Surgery, University Medical Center Utrecht, Heidelberglaan 100, Utrecht, The Netherlands; Department of Surgery, University Medical Center Utrecht, Heidelberglaan 100, Utrecht, The Netherlands; Department of Surgery, University Medical Center Utrecht, Heidelberglaan 100, Utrecht, The Netherlands; Department of Surgery, University Medical Center Utrecht, Heidelberglaan 100, Utrecht, The Netherlands

**Keywords:** anastomotic leakage, esophagectomy, Robotic surgery, treatment

## Abstract

Anastomotic leakage is a feared complication after esophagectomy and associated with increased post-operative morbidity and mrotality. The aim of this study was to evaluate the management of leakage after robot-assisted minimally invasive esophagectomy (RAMIE) with intrathoracic anastomosis. From a single center prospectively maintained database, all patients with anastomotic leakages defined by the Esophageal Complications Consensus Group between 2016 and 2021 were included. Contained leakage was defined as presence of air or fluid at level of the anastomosis without the involvement of the mediastinum or thorax. Non-contained leakage was defined as mediastinitis and/or mediastinal/pleural fluid collections. The primary outcome was 90-day mortality and the secondary outcome was successful recovery. In this study, 40 patients with anastomotic leakage were included. The 90-day mortality rate was 3% (*n* = 1). Leakage was considered contained in 29 patients (73%) and non-contained in 11 patients (27%). In the contained group, the majority of the patients were treated non-surgically (*n* = 27, 93%) and management was successful in 22 patients (76%). In the non-contained group, all patients required a reoperation with thoracic drainage and management was successful in seven patients (64%). Management failed in 11 patients (28%) of whom 7 developed an esophagobronchial fistula, 3 had a disconnection of the anastomosis and 1 died of a septic bleeding. In conclusion, this study demonstrates that the management anastomotic leakage in patients who underwent RAMIE with an intrathoracic anastomosis was successful in 73% of the patients with a 90-day mortality rate of 3%. A differentiated approach for the management of intrathoracic anastomotic leakage is proposed.

## INTRODUCTION

Esophagectomy is the cornerstone of treatment for patients with locally advanced esophageal cancer.^1^ Recently, there has been a vast increase in the use of minimally invasive techniques for esophagectomy as they have been associated with less post-operative complications and comparable oncological outcomes compared to open esophagectomy.^2–4^ Despite technical improvements, esophageal surgery remains a highly complex procedure associated with significant post-operative morbidity.^5^

Anastomotic leakage after esophagectomy is one of the most feared post-operative complication and associated with significant morbidity, prolonged hospital stay, increased healthcare expenditure and reduced (long-term) survival.^6–8^ Treatment of anastomotic leakage is challenging and standardized treatment protocols are lacking.^9–12^ The manifestation and primary treatment strategy of leakage largely depends on the location of the anastomosis. Although the reported incidence of anastomotic leakage is higher for a cervical anastomosis compared to an intrathoracic anastomosis, leakage of an intrathoracic anastomosis has been associated with more severe intrathoracic manifestations requiring a different treatment strategy.^13^^,^^14^

In our center, a robot-assisted hand-sewn intrathoracic anastomosis was first implemented in 2016 and the anastomotic technique was further improved during the following years.^15^^,^^16^ During this period, different manifestations of anastomotic leakage were observed requiring different treatment strategies. The aim of this study was to evaluate the treatment of patients with anastomotic leakage after robot-assisted minimally invasive esophagectomy (RAMIE) with an intrathoracic anastomosis and to present a differentiated approach to manage intrathoracic anastomotic leakage.

## METHODS

### Patient population

Patients were selected from a prospectively maintained institutional database. In the database, patient characteristics, details of the surgical procedure and complications were prospectively registered during a weekly multidisciplinary meeting. Patients who underwent RAMIE with intrathoracic anastomosis in the University Medical Center Utrecht (UMC Utrecht) and developed anastomotic leakage were extracted from the database and included in the study. All esophagectomies with intrathoracic anastomosis are performed robotically in the UMC Utrecht. As the intrathoracic robot-assisted hand-sewn anastomosis was introduced in February 2016, patients were included from February 2016 to December 2021. There were no exclusion criteria. The institutional review board approved this study and the need for written informed consent was waived.

### Surgical technique and post-operative management

RAMIE consisted of a field-field lymphadenectomy and creation of a gastric conduit followed by a hand-sewn intrathoracic anastomosis. A detailed description of the procedure was published previously.^17^ In summary, the anastomoses were created as follows: indocyanine green was used to assess the perfusion of the gastric conduit and determine the most optimal location for the anastomosis. In case of mal perfusion of the conduit tip, this part was stapled off. The anastomoses were created by a hand-sewn, end-to-side, technique with a barbed 4/0 running V-Loc (Medtronic/Covidien; Minneapolis, MN, USA). From April 2018 onwards, three to four tension releasing stiches with Vicryl 3/0 (Ethicon, Somerville, NJ) were added to the technique to reduce the traction on the anastomosis. An omental wrap concluded the anastomosis. All patients who underwent RAMIE were treated according to our local enhanced recovery after esophagectomy (EROES) protocol. Components of the EROES protocol included preoperative optimization by a dietician and physiotherapist, epidural or paravertebral analgesia, direct extubation in the operation room and early mobilization. During the study period several adjustment to the EROES protocol were made. Initially, all patients resumed oral intake immediately after surgery. From January 2019 onwards, all patients received a feeding jejunostomy whilst being kept on a nil by mouth diet. On the fourth post-operative day a contrast swallow was performed. The primary aim of the contrast swallow was to asses for delayed conduit emptying. If there were no signs of delayed gastric emptying, patients were allowed to start oral water intake. In the absence of post-operative complications, solid food was allowed from the 14th post-operative day and onwards.

### Anastomotic leakage definitions

Anastomotic leakage was defined according to the Esophageal Complications Consensus Group (ECCG).^18^ Anastomotic leakage was suspected when a patient had a combination of signs including fever, tachycardia, elevated serum levels of C-reactive protein (CRP) and/or elevated leucocytes. When anastomotic leakage was suspected, patients underwent a computed tomography (CT) scan and an additional endoscopy if needed. A contrast swallow examination was not used to detect anastomotic leakage as its predictive value is too low.^19^^,^^20^ In addition, it does not provide information on mediastinal or pleural involvement. At the day of diagnosis, anastomotic leakage was considered contained or non-contained based on a CT-scan. Leakage was defined as contained in case of local presence air or fluid at level of the anastomosis, without the involvement of the mediastinum or thoracic cavity. Non-contained leakage was defined as presence of mediastinitis and/or mediastinal/pleural fluid collections. All CT-scans performed at the day of diagnosis were retrospectively reviewed and scored and scored in a consensus as contained or non-contained leak (EdG, SB, JB, SH and RvH). Observers were blinded for the patients’ outcome. For the current analysis, the score was not adjusted in case a leak developed from a contained leak into a non-contained leak after the day of diagnosis.

### Management of anastomotic leakage

When anastomotic leakage was suspected, all patients were immediately treated with a nil by mouth regimen, endoscopic placement of a nasogastric tube in the gastric conduit and intravenous antibiotics. Additionally, depending on the severity of anastomotic leakage, patients were treated endoscopically and/or surgically. Endoscopic treatment consisted of placement of a suction-drain placed through the anastomotic defect into the mediastinum or placement of a stent. Surgical treatment consisted of drainage and washing of the thoracic cavity by a video-assisted thoracoscopy or thoracotomy. When the anastomosis was completely dehiscent during the reoperation, or when there was severe gastric conduit necrosis, the anastomosis had to be disconnected. Gastric conduit necrosis was diagnosed intraoperatively based on macroscopic appearance of the conduit combined with the endoscopically observed appearance of the mucosa and transmural defect.

### Follow-up

After hospital discharge, all patients with anastomotic leakage visited the outpatient clinic and underwent a contrast swallow investigation to evaluate anastomotic integrity. In some cases, a CT-scan or endoscopy was performed to evaluate anastomotic integrity. If the anastomosis was deemed sufficient, patients restarted their oral intake. Hereafter, patients were routinely followed at the outpatient clinic every 3 months in the first year, every 6 months during the second year and yearly until 5 years after RAMIE.

### Outcomes

The primary outcome was 90-day mortality. The secondary outcome was successful recovery after treatment for anastomotic leakage. Recovery was defined to be successful when the following criteria were met:

- The defect in the anastomosis healed, confirmed by radiology (CT-scan/contrast swallow) or endoscopy leading to restart oral intake.
- The patient did not develop an esophagobronchial/esophagopleural fistula during the follow-up.
- No disconnection of the anastomosis was performed.

Other outcomes were as follows: days between RAMIE and diagnosis of anastomotic leakage, diet at moment of diagnosis, serum levels of CRP and leucocytes measured at the day of diagnosis, pneumonia defined by the Uniform Pneumonia Score,^21^ in-hospital mortality, duration of hospital stay, duration of intensive care unit (ICU) stay and readmissions to the ICU. In addition, complications, such as esophagobronchial fistula and anastomotic strictures defined by the need of endoscopic dilation were reported.

### Statistical analysis

For the purpose of this study, only descriptive analyses were performed. Categorical variables were demonstrated as count with percentage. Continuous variables were demonstrated as mean (with standard deviation) or median (with range), depending on the type of data. All statistical analyses were performed by using SPSS 25.0 (IBM).

## RESULTS

### Study population

Between February 2016 and December 2021, 152 patients underwent RAMIE with an intrathoracic anastomosis after which 40 patients developed anastomotic leakage (26%) whom were included in this study. There were no differences in patient characteristics of both patients with and without anastomotic leakage (Table 1). Of the 40 patients with anastomotic leakage, 29 (73%) were classified as contained and 11 (27%) as non-contained leaks at the day of diagnosis. Anastomotic leakage was diagnosed after a median of 8 days (range 1–22) post-operatively. At diagnosis, 22 (55%) patients were on a liquid diet, 17 (43%) patients on a nil by mouth policy and 1 (3%) patient had intake for solid food. On the day of diagnosis, the median serum level of CRP was 188 (range 38–404) and of median leucocytes was 13.8 (range 5.1–26.4).

**Table 1:** Baseline characteristics of patients with and without anastomotic leakage

Variables	Anastomotic leakage (*n* = 40)	No anastomotic leakage (*n* = 112)
**Age (median, range)**	64 (39–78)	66 (42–81)
**BMI (median, range)**	26 (19–43)	25 (16–36)
**Gender** Male	35 (88%)	84 (75%)
**Comorbidities**	32 (80%)	80 (71%)
**ASA-score** 1 2 3 4	3 (8%) 22 (55%) 15 (38%)	12 (11%) 68 (61%) 29 (26%) 1 (1%)
**Neoadjuvant therapy** Chemoradiotherapy None Chemoterhapy	37 (93%) 3 (8%)	100 (89%) 8 (7%) 4 (3%)
**Clinical T Stage** cT1 cT2 cT3 cT4	3 (8%) 10 (25%) 25 (63%) 2 (5%)	7 (6%) 19 (17%) 81 (72%) 5 (5%)

### Primary outcome

One patient in the non-contained group died after discharge within 90 days after RAMIE due to a septic bleeding. The septic bleeding was most likely caused by an ongoing anastomotic leakage that led to an abscess that damaged the aortic wall, resulting in a rupture.

### Management of contained anastomotic leakage

**Fig. 1:**
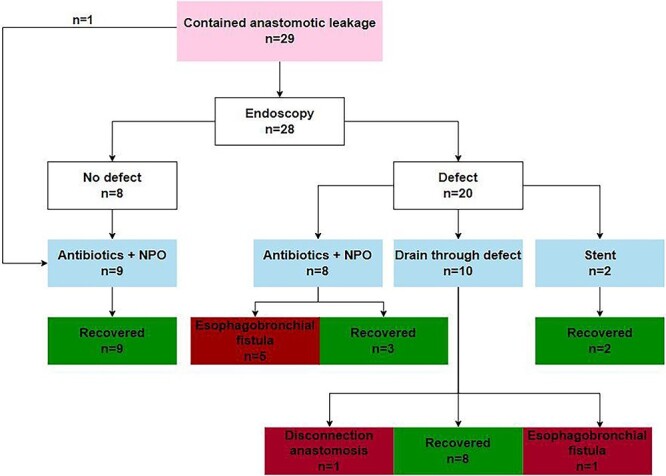
Evaluation of the management of 29 patients with a contained anastomotic leak.

The majority of patients had a contained anastomotic leak (*n* = 29, 73%). Management of anastomotic leakage in these patients is summarized in Figure 1.

In 28 of the 29 patients, an endoscopy was performed to evaluate the integrity of the anastomosis. In one patient, no endoscopy was performed and anastomotic leakage was diagnosed based on radiological findings. This patient was treated with antibiotics and a nil by mouth diet after which the patient recovered successfully.

A visible anastomotic defect was present in 20 of the 28 patients who underwent endoscopy. The eight patients without a visible defect were kept on a nil by mouth diet and received intravenous antibiotics. They all recovered successfully.

Of the 20 patients with a visible anastomotic defect, 10 patients were treated with a drain through the defect into the mediastinum combined with antibiotics and a nil by mouth diet, 8 patients with antibiotics and a nil by mouth diet only and 2 patients with an endoscopically placed stent. Of the 10 patients that were treated with a drain through the anastomotic defect, 8 recovered successfully, 1 developed an esophagobronchial fistula and the anastomosis had to be disconnected due to complete dehiscence in 1 patient. Of the eighy patients who were only treated with antibiotics and a nil by mouth diet, five developed an esophagobronchial fistula and three patients recovered successfully. The two patients treated with an endoscopically placed stent recovered successfully.

In summary, 22 out of 29 patients (76%) with a contained leak recovered successfully. A healed anastomosis was observed after a median of 30 days (range 16–78) after the diagnosis of leakage. Treatment failed in seven (24%) patients; in one patient, the anastomosis was disconnected due to complete dehiscence, and six patients developed an esophagobronchial fistula. Of the six patients who developed an esophagobronchial fistula, five patients had a visible anastomotic defect during endoscopy and were initially treated with antibiotics and a nil by mouth diet only for their anastomotic leakage.

### Management of non-contained anastomotic leakage

**Fig. 2:**
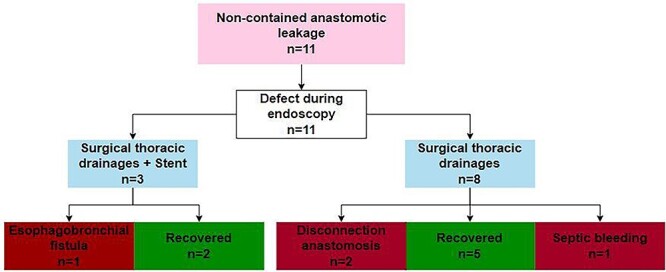
Evaluation of the management of 11 patients with a non-contained anastomotic leak.

Of the 40 patients, 11 (27%) were classified as having non-contained anastomotic leakage. Management of these patients is summarized in Figure 2. Treatment consisted of surgical thoracic drainage in all patients (*n* = 11) of which three patients received an endoscopic stent simultaneously. Treatment was successful in seven patients, the anastomosis had to be disconnected due to severe leakage in two patients, one patient developed an esophagobronchial fistula and one patient died after discharge due to a septic bleeding, as mentioned previously.

In summary, 7 out of 11 patients with a non-contained leak (64%) recovered successfully with a healed anastomosis after a median of 51 days (range 25–88) from the day of diagnosis.

### Clinical outcomes

Clinical outcomes are presented in Table 2. The median hospital stay was 22 days (range 5–139) and the median stay on the ICU was 1 day (range 0–72) with a readmission rate to the ICU of 38% (*n* = 15). There was no in-hospital mortality. The median follow-up was 41 months (range 1–60). An esophagobronchial fistula occurred in 7 (18%) patients, median 58 days after RAMIE (range 15–698). These fistulas were treated endoscopically in four patients and surgically in three patients. Endoscopic treatment consisted of stent placement in three patients and an endoscopic clip in one patient. Surgical treatment consisted of bronchial repair with a bovine patch followed by interposition of healthy tissue in all three patients. Strictures were diagnosed in 19 (48%) patients after a median of 94 days following RAMIE (range 41–383). Strictures were treated with endoscopic dilatations in all patients with a median of 5 dilatations (range 1–20). The stricture rate for patients without anastomotic leakage was 47% (*n* = 53). Strictures were treated with endoscopic dilatations in all patients with a median of four dilatations (range 1–67).

**Table 2:** Clinical outcomes of 40 patients with anastomotic leakage after an intrathoracic anastomosis

Variables	*N* = 40
Esophagobronchial fistula	7 (18%)
Strictures	19 (48%)
In-hospital mortality	0 (0)
90-day mortality	1 (3%)
Duration in hospital stay, days (median, range)	22 (5–139)
Duration ICU stay, days (median, range)	1 (0–72)
Readmission to the ICU	15 (38%)

## DISCUSSION

This study evaluated the treatment strategy of anastomotic leakage in 40 patients who underwent RAMIE with an intrathoracic anastomosis. Anastomotic leakage was considered contained in 29 patients of which the majority was treated non-surgically (*n* = 27, 93%). In this group, treatment was successful in 22 patients (76%). The other 11 patients had a non-contained leak with thoracic manifestation who all required a reoperation. Treatment was successful in seven patients (64%). No in-hospital mortality occurred and the 90-day mortality rate was 3% (*n* = 1). Failures were mainly due to inadequately drained leaks in the contained group, resulting in an esophagobronchial fistula in five patients.

Previously reported success rates in literature of different treatment strategies for anastomotic leakage range between 50 and 100%.^11^ However, it is hard to compare success rates between studies as the limited number of studies on this topic have a small sample size (range 5–28) and used different definitions for successful treatment of anastomotic leakage. As proxy for the success of treatment of the anastomotic leakage, 90-day mortality rates are often used. In this context, a recent international multicenter study that included 319 patients with anastomotic leakage reported a 90-day mortality rate of 12%.^13^ Other studies have reported similar 90-mortality rates after anastomotic leakage ranging between 10% and 35%.^11^^,^^22^ The 3% 90-day mortality after anastomotic leakage in the current study is considerably lower and therewith supports the proposed treatment strategy for anastomotic leakage.

**Fig. 3:**
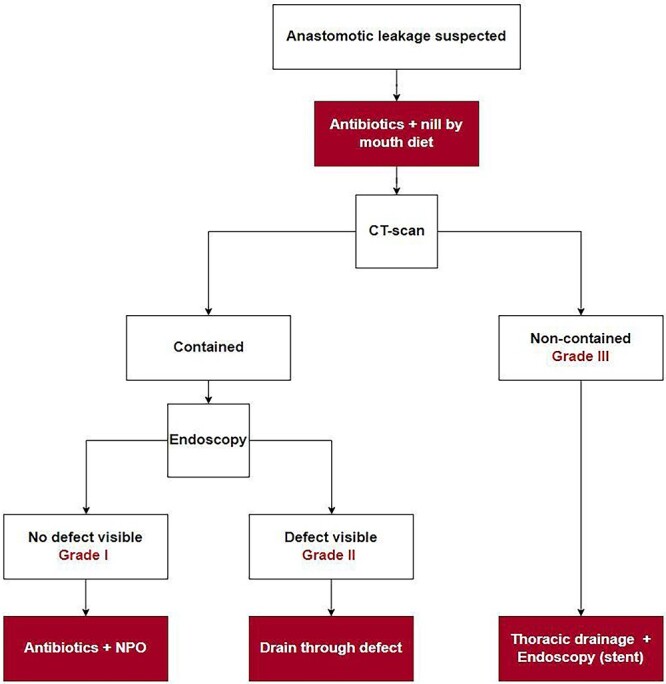
Differentiated approach for the management of anastomotic leakage after RAMIE with an intrathoracic anastomosis.

Despite the low mortality rate, management of leakage failed in 11 patients (28%) of which the majority developed an esophagobronchial fistula. In this study, five of eight patients with a visible anastomotic defect during endoscopy treated with antibiotics only developed an esophagobronchial fistula. The high rate of esophagobronchial fistula in these patients is likely caused by inadequately drained anastomotic leakage after which the persistent abscess between the esophagus and bronchus developed into an esophagobronchial fistula. Based on these results, a differentiated approach for the management of anastomotic leakage was developed and is presented in Figure 3. Essential in this strategy is the importance of drain placement in case of a visible anastomotic defect during endoscopy. Therefore, we recommend that all patients with anastomotic leakage, even if considered contained, should undergo an endoscopy to evaluate the integrity of the anastomosis. If a defect is visible, a nasogastric drain is placed through the defect into the mediastinum. Following this strategy, no more esophagobronchial fistulas were diagnosed from January 2019 onwards. Furthermore, management of a non-contained leaks should always consist of a reoperation with thoracic drainage. In addition, an endoscopy should be performed in order to create source control with a stent or additional drainage through the anastomotic defect.

The role of a stent in the management of anastomotic leakage is still under debate in literature.^23^ In our opinion, a stent could be used in non-contained leaks to provide source control combined with pleural or mediastinal drainage. On the other hand, stents are associated with failure and complications such as migration of the stent, which occurs in 20% of the patients.^24^ An alternative might be the use of an endoscopic vacuum-assisted closure (Endovac (B. Braun, Melsungen, Germany)) device with reported success rates of 60%–80%.^25–28^ Higher negative pressure could be achieved with an Endovac compared with a drain through the anastomotic defect into the mediastinum. However, whether the Endovac achieves better results over a drain through the mediastinum is still unclear as high quality studies are scarce. In addition, serious disadvantages are reported as well, including Endovac migration, bleeding, trachea damage and the need for multiple endoscopies to change the Endovac device, which is burdensome for patients and logistically difficult.^26^^,^^29^

The incidence of anastomotic leakage was relatively high in this cohort. This is most likely caused by a learning curve effect for the hand-sewn robot-assisted intrathoracic anastomosis, that has been published previously.^15^^,^^16^ In the most recent 1.5 years, our leak rate decreased to 6%, indicating that proficiency is being reached.

The severity grade of anastomotic leakage according to the ECCG is currently based on the type of treatment (conservative, endoscopic, surgical). Although the current definition allows for an objective classification, a definition to classify severity based on leakage characteristics might be more helpful for applying and providing recommendations for the right treatment. In addition, if a center would treat leakages more frequently endoscopically, the severity grade of anastomotic leakage of that center will decrease while the nature of the leakage is in fact not changing. Therefore, we recommend a new classification that is based on radiological and endoscopic findings to define the severity of anastomotic leakage. Based on radiology, anastomotic leakage should be defined as contained (grade I or II) or non-contained (grade III) according to the definition used in this study. Based on endoscopy, a contained leak could be classified as grade I when no defect is visible or grade II in case of a visible anastomotic defect.

A strength of this study is the large cohort of patients treated with a uniform RAMIE technique and an unique level of detail regarding treatment strategies for anastomotic leakage. In addition, the anastomotic leakage was scored prospectively. A limitation of this study is that developments in perioperative care might have caused a heterogeneous cohort. However, bias caused by a heterogeneous cohort should be minimal as the aim of this study was to evaluate the treatment strategy of leakage.

In conclusion, this study demonstrated that the management of anastomotic leakage in patients who underwent RAMIE with intrathoracic anastomosis was successful in more than 70% of the patients with an in-hospital mortality of 0% and a 90-day mortality of 3%. A visible anastomotic defect during endoscopy always requires drainage by endoscopic drain placement through the defect or surgical thoracic drainage. Based on these findings, a differentiated approach for the management of intrathoracic anastomotic leakage is proposed.
